# Persistent Asymptomatic Pneumoperitoneum With Spontaneously Resolving Idiopathic Pneumatosis Intestinalis: A Case Report

**DOI:** 10.1155/cris/5524896

**Published:** 2025-06-04

**Authors:** John P. Ratanawong, Tzu Han Huang, Torben H. Urdahl, Katherine Weir, Anthony T. Rezcallah

**Affiliations:** ^1^Department of Surgery, University of Minnesota, Minneapolis, Minnesota, USA; ^2^Department of Surgery, Minneapolis Veteran's Affairs Medical Center, Minneapolis, Minnesota, USA

## Abstract

We present our experience with a patient with acute-on-chronic asymptomatic pneumoperitoneum with spontaneously resolving idiopathic pneumatosis intestinalis that was solely managed on close observation alone. This case is unique in that it details the approach to nonoperative management of massive free air under the diaphragm identified incidentally on routine preventative health screening and longitudinal follow-up over an 8-month period. In the absence of known and underlying systemic disease, efficient and coordinated clinical work-up and evaluation for comorbid diagnoses associated with pneumoperitoneum can serve to guide management and avoid unnecessary surgery for stable and asymptomatic patients.

## 1. Introduction

Massive free air in the abdomen or pneumoperitoneum is an acute radiographic finding that can be associated with life-threatening diagnoses, often requiring immediate operative management. Very seldom is spontaneous pneumoperitoneum found to have a benign etiology, as most symptomatic cases are attributable to infectious, iatrogenic, inflammatory, and other causes that ultimately lead to an acute abdomen [1]. Cases of asymptomatic, idiopathic pneumoperitoneum due to pneumatosis intestinalis or intramural gas found within the bowel wall are even more rare. To date, there have been only a handful of cases of pneumoperitoneum secondary to underlying pneumatosis intestinalis in the published literature. Here, we describe a patient who presented with asymptomatic pneumoperitoneum incidentally found on radiographic imaging and also idiopathic small bowel pneumatosis that spontaneously resolved with observation alone.

## 2. Case Presentation

**Figure 1:**
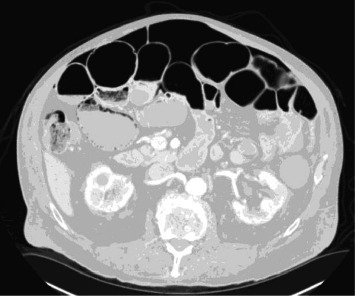
Low-dose CT images for lung nodule surveillance protocol demonstrating massive free air underneath the diaphragm.

An 84-year-old man with a history of heart failure with preserved ejection fraction, chronic kidney disease, and chronic obstructive pulmonary disease presented to the radiology outpatient department for low-dose CT imaging for routine lung nodule surveillance. After the patient was sent home, the reviewed images demonstrated incidental massive free air within the upper abdomen, resulting in the staff radiologist calling the patient informing him that he needed to return to the emergency department (Figure 1). He did not report any recent episodes of acute abdominal pain, nausea, vomiting, shortness of breath, chest pain, or changes in bowel or bladder habits.  Table 1 provides a brief summary of his medical history, surgical history, and medications.

**Figure 2:**
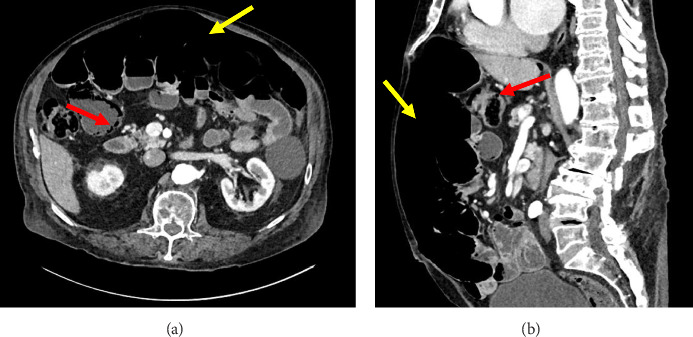
Initial ED presentation CT abdomen and pelvis with contrast: (A) Axial view demonstrating large anterior pneumoperitoneum (yellow arrow) with pneumatosis intestinalis (red arrow) present in a dependent portion of the small intestine. (B) Sagittal view demonstrating large anterior pneumoperitoneum (yellow arrow) spanning from the distal-most aspect of the abdomen to the diaphragm. Small pneumatosis intestinalis (red arrow) is also present in a dependent portion of the small intestine.

In the emergency department, he was afebrile with vital signs within normal limits and benign findings on examination. In particular, mild abdominal distension and diastasis recti were noted on abdominal examination that was otherwise unremarkable for any acute changes concerning for peritonitis and small bowel obstruction in the setting of volvulus, appendicitis, or hernias. Likewise, his laboratory testing, which included a comprehensive metabolic panel and complete blood count, was also nonconcerning. The patient reported his last bowel movement was earlier that morning. At that time, he was given prophylactic intravenous piperacillin/tazobactam in case of a possible bowel perforation. Following this, a CT abdomen/pelvis was obtained which identified significant free air within the anterior abdomen and pneumatosis intestinalis of several dilated loops of small bowel in the right mid-abdomen. Other findings included a mild twisting of the mesentery, redundant distal small bowel loops, and a normal appearing colon (Figure 2). Given his clinically asymptomatic status and negative history of prior abdominal or endoscopic surgeries, he was subsequently admitted for observation with possible exploratory laparotomy if the patient demonstrated signs of decompensation. He was later discharged after an uneventful 2-day hospital stay, where he tolerated a regular diet, independent ambulation, and spontaneous voiding. He was then scheduled for monthly outpatient follow-up with general surgery.

**Figure 3:**
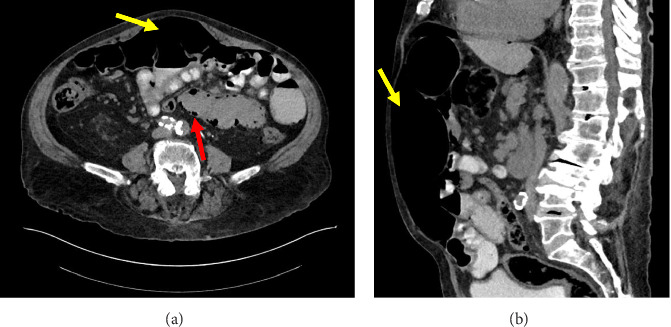
Four-week repeat CT abdomen and pelvis with contrast upon repeat ED presentation: (A) Axial view demonstrating persistent pneumoperitoneum (yellow arrow) and persistent pneumatosis intestinalis (red arrow) of a portion of the small intestine. (B) Sagittal view demonstrating persistent pneumoperitoneum (yellow arrow) spanning from the distal-most aspect of the abdomen to the diaphragm.

One month after his initial encounter, the patient presented back to the emergency department to ask questions about the etiology of his unexplained pneumoperitoneum. He denied any changes in his clinical status since his last outpatient follow-up and continued to be clinically asymptomatic with benign laboratory testing and a nonsurgical abdomen on exam. Repeat CT imaging redemonstrated the existing pneumoperitoneum, pneumatosis, and dilated loops of small bowel with intact mesenteric vasculature (Figure 3). After discussing the findings with no clear underlying cause for his presentation, we recommended further in-patient observation. The patient declined to be admitted, but he was cautioned to return if experiencing new onset bowel symptoms. He had regular follow-ups afterward at 1-month and 6-month intervals, with no change in his asymptomatic condition.

**Figure 4:**
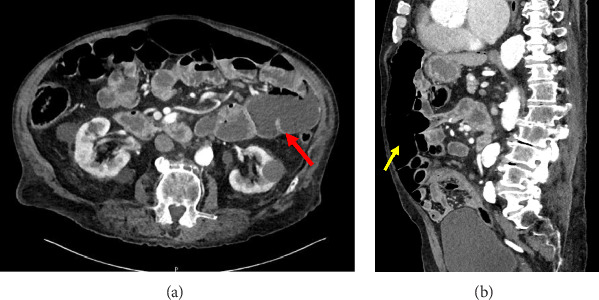
Repeat CT abdomen and pelvis at 8-month follow-up visit: (A) Axial view demonstrating improved pneumoperitoneum and no signs pneumatosis intestinalis, therefore, indicating resolution (red arrow). (B) Sagittal view demonstrating improved pneumoperitoneum (yellow arrow) compared to previous CT scans.

Eight months after his initial presentation, he returned to the hospital with acute onset epigastric “spasm-like” abdominal pain that worsened after a bowel movement. On CT imaging, he still had free air within the upper abdomen, although less than 8 months prior. There was also complete resolution of the LUQ small bowel loop pneumatosis, but with new fluid-filled small bowel dilations, as well as areas concerning for possible stricture (Figure 4). His benign physical examination was reassuring for no acute surgical emergency and improving benign pneumoperitoneum. He was discharged after 3 days of observation for concomitant generalized weakness and continued to follow-up with his primary care provider. His abdomen remains nonsurgical and he continues to have asymptomatic pneumoperitoneum without known recurrence of his pneumatosis on conservative management only.

## 3. Discussion

Spontaneous idiopathic pneumoperitoneum in the setting of benign pneumatosis intestinalis has shown to be a difficult clinical problem to investigate, as the majority of the literature consists of case reports with few robust studies. This is mainly due to the extreme rarity of encountering both extraluminal free air that is spontaneous as well as pneumatosis intestinalis in a benign abdomen. Notably, there has only been one published report that closely matches the presented case. However, in that report, the patient's persisting pneumoperitoneum with resolved pneumatosis was linked to chronic systemic sclerosis, a known cause of both of these phenomena [2]. A study by Adachi et al. [3] observed similar cases, but with pneumatosis resolving within 7 days on follow-up CT and persistent extraluminal free air linked to bowel perforation, not idiopathic causes. Thus, to the best of our knowledge, we believe this is the first reported case of completely asymptomatic longstanding pneumoperitoneum in the setting of benign idiopathic pneumatosis intestinalis that spontaneously resolved with observation.

Table 2 outlines the known etiologies of both pneumatosis intestinalis and pneumoperitoneum [1, 4–8]. The pathogenesis of pneumatosis intestinalis is unclear, although there have been several theories devised, including mechanical, bacterial, pulmonary, and chemical [6, 7]. The mechanical theory states that increased intraluminal pressure from mechanical disturbances (e.g., trauma, gastrointestinal procedures, and bowel obstruction) can lead to gas intrusion into the intestinal wall. The bacterial theory suggests that gas-producing gut microbes can translocate into intestinal wall and produce excessive hydrogen gas causing pneumatosis intestinalis. The pulmonary theory hypothesizes that pulmonary diseases (e.g., asthma and COPD) can result in alveoli rupture and air entering the mediastinum that then dissects along the aorta, into the mesenteric vessels, and ultimately end up in the intestinal walls. Last, the chemical theory proposes that malnutrition can lead to decrease in carbohydrate breakdown and increased bacterial fermentation in the bowel, producing excessive gas byproducts [9]. With respect to our patient, the pulmonary theory is the most applicable to understand the driving forces behind his unique presentation, given that his medical history is notable for COPD and no other systemic disease. However, it remains unclear how much of his presentation can be explained by his pulmonary disease alone, since this is the first documented case of spontaneous pneumoperitoneum with benign pneumatosis intestinalis in the absence of any systemic disease or identifiable cause.

Despite an exceptionally unique presentation, timely recognition of massive free air by the radiologists allowed our patient to be quickly recalled back for urgent evaluation from the outpatient setting and community. This decision resulted in efficient coordination of care between radiology, emergency medicine, and surgery to both stabilize and monitor the patient for signs of acute decompensation and the need for emergent intervention. While the patient never reached acute status, this enabled the care team to establish a baseline and readily follow his case over the course of 8 months from his initial presentation on medical management alone. This patient's asymptomatic presentation in the absence of an acute abdomen and its chronicity documented over several months provide a helpful framework to inform clinical decision-making in determining the appropriateness for urgent or emergent surgical intervention versus careful observation with close follow-up and routine monitoring. This is especially useful when encountering such an exceedingly rare combination of both asymptomatic pneumoperitoneum and benign pneumatosis intestinalis without any known underlying risk factors.

## 4. Conclusions

Chronic asymptomatic massive pneumoperitoneum with eventual spontaneous resolution of pneumatosis intestinalis in a patient with a nonsurgical abdomen is rare. When incidentally found and the chronicity is not yet appreciated, this condition requires timely and coordinated evaluation and workup in the urgent setting given the possible surgical causes. In the absence of an acute surgical abdomen and known, underlying systemic disease, massive pneumoperitoneum can be successfully approached with expectant management and the flexibility to shift to operative management, should the patient's course mandate it.

## Figures and Tables

**Table 1:** Summary of active medical problems, medications, surgical history, and social history.

Active medical problems	Diabetes mellitus type 2, hypertension, congestive heart failure, hyperlipidemia, chronic obstructive pulmonary disease, paroxysmal atrial fibrillation, thoracic aortic aneurysm without rupture, insomnia, chronic kidney disease, gout, aortic valve stenosis, interstitial lung disease
Medications	Carboxymethylcellulose, mupirocin, hydrocodone/acetaminophen, torsemide, allopurinol, loperamide, atorvastatin, fluticasone/salmeterol INH, albuterol INH, prednisone, apixaban, calcitriol, acetaminophen, loratadine, omega 3 liquid, magnesium oxide tablet, vitamin B complex supplement, amiodarone, trazodone
Social history	Tobacco: former smoker (quit ≥25 years ago); alcohol: history of abuse (quit ≥40 years ago)

**Table 2:** Side-by-side comparison of the etiologies of spontaneous pneumoperitoneum and pneumatosis intestinalis organized by system [1, 4–8].

Pneumatosis intestinalis		Pneumoperitoneum
Primary causes	Pneumatosis cystoides intestinalis (occurs in 15% cases) Idiopathic	Idiopathic

Secondary causes	Ischemia Bowel obstruction*⁣*^*∗*^ Volvulus*⁣*^*∗*^ Malrotation*⁣*^*∗*^ Intussusception*⁣*^*∗*^ Mesenteric vascular disease*⁣*^*∗*^ Inflammatory Inflammatory bowel disease Appendicitis Diverticular disease Toxic megacolon*⁣*^*∗*^ Infectious Viral (HIV, CMV, rotavirus) Bacterial (*Clostridium difficile*, Klebsiella, Escherichia) Fungal (Cryptosporidium) Enteritis*⁣*^*∗*^ Colitis*⁣*^*∗*^ Whipple disease Intestinal parasites Systemic disease Scleroderma Systemic lupus Liver failure Pulmonary disorders COPD Asthma Bronchitis Pulmonary fibrosis Positive end expiratory pressure Cystic fibrosis Iatrogenic Endoscopy Drug-induced (e.g., chemotherapy, barium enema, corticosteroid, lactulose, sorbitol, voglibose) Surgical anastomosis Jejunoileal bypass Jejunostomy tube Immunodeficiency AIDS Graft versus host disease Organ transplant*⁣*^*∗*^ Malignancy Leukemia Other Peptic ulcer disease Pyloric stenosis Malnutrition Blunt trauma*⁣*^*∗*^ Pseudo-obstruction Ingestion of corrosive agents*⁣*^*∗*^	Gastrointestinal Gastrointestinal perforation (90% cases) Pneumatosis cystoides intestinalis Collagen vascular disease pneumocholecystitis Jejunal and sigmoid diverticulosis distended hollow viscus Subclinical perforated viscus Intrathoracic Blunt thoracic trauma Barotrauma Post-cardiopulmonary resuscitation excessive mechanical ventilation Pneumothorax Pneumomediastinum Gynecological Pelvic inflammatory disease Pelvic exam Sexual intercourse Vaginal douching Vaginal insufflation Iatrogenic Laparoscopy Laparotomy Gastrointestinal endoscopy Pelvic endoscopy Peritoneal dialysis/paracentesis Others Jacuzzi usage Scuba diving

*Note*: *⁣*^*∗*^ indicates life-threatening causes.

## Data Availability

The data that support the findings of this study are available from the corresponding author upon reasonable request.
